# Supplementary material for: Comparative multiomics analysis of cell physiological state after culture in a basket bioreactor
Source: Sci Rep. 2022 Nov 23;12:20161. doi: 10.1038/s41598-022-24687-4 (PMC9686226; doi:10.1038/s41598-022-24687-4)
Supplement: Supplementary file 1 — Supplementary Information 1. [file 41598_2022_24687_MOESM1_ESM.zip › raw data/Proteomic raw data/4.DiffExprAnalysis/EG.vs.CG/EG.vs.CG.volcano.pdf]

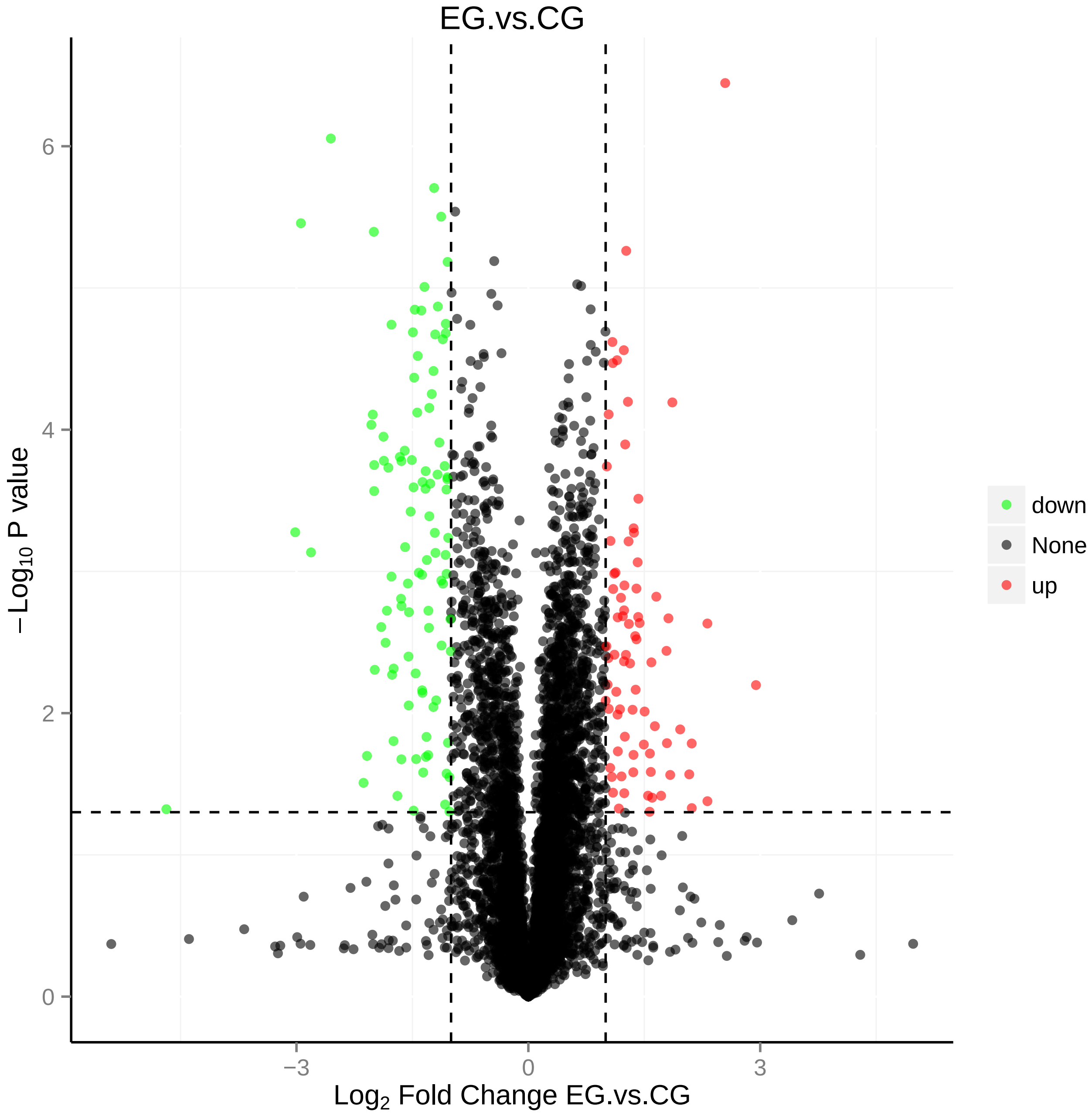

EG.vs.CG
$-Log_{10}$ P value
6
4
2
0
−3
0
3
Log_2 Fold Change EG.vs.CG
down
None
up
